# The Correlation Between Olfactory Test and Hippocampal Volume in Alzheimer's Disease and Mild Cognitive Impairment Patients: A Meta-Analysis

**DOI:** 10.3389/fnagi.2021.755160

**Published:** 2021-10-20

**Authors:** Ming-Wan Su, Jing-Nian Ni, Tian-Yu Cao, Shuo-Shi Wang, Jing Shi, Jin-Zhou Tian

**Affiliations:** ^1^Dongzhimen Hospital, Beijing University of Chinese Medicine, Beijing, China; ^2^Department of Neurology, Dongzhimen Hospital, Beijing University of Chinese Medicine, Beijing, China

**Keywords:** olfactory deficits, hippocampus, mild cognitive impairment, Alzheimer's disease, meta-analysis

## Abstract

**Background:** Previous studies have reported that olfactory identification deficits may be the earliest clinical features of Alzheimer's disease (AD). However, the association between odor identification and hippocampal atrophy remains unclear.

**Objective:** This meta-analysis quantified the correlation between odor identification test scores and hippocampal volume in AD.

**Method:** A search of the PUBMED, EMBASE, and WEB OF SCIENCE databases was conducted from January 2003 to June 2020 on studies with reported correlation coefficients between olfactory identification score and hippocampal volume in patients with amnestic AD or mild cognitive impairment (MCI). The quality of the studies was assessed using the Newcastle-Ottawa quality assessment scale (NOS). Pooled *r-*values were combined and computed in R studio.

**Results:** Seven of 627 original studies on AD/MCI using an olfactory identification test (*n* = 902) were included. A positive correlation was found between hippocampal volume and olfactory test scores (*r* = 0.3392, 95% CI: 0.2335–0.4370). Moderator analysis showed that AD and MCI patients were more profoundly correlated than normal controls (AD: *r* = 0.3959, 95% CI: 0.2605–0.5160; MCI: *r* = 0.3691, 95% CI: 0.1841–0.5288; NC: *r* = 0.1305, 95% CI: −0.0447–0.2980). Age difference and patient type were the main sources of heterogeneity in this analysis.

**Conclusion:** The correlation appears to be more predominant in the cognitive disorder group (including MCI and AD) than in the non-cognitive disorder group. Age is an independent factor that affects the severity of the correlation during disease progression. The mildness of the correlation suggests that olfactory tests may be more accurate when combined with other non-invasive examinations for early detection.

**Systematic Review Registration:** https://inplasy.com/, identifier INPLASY202140088.

## Introduction

Alzheimer's disease (AD) is an insidiously progressive neurodegenerative disease that primarily causes dementia. It is estimated that 44 million people live with this condition (Lane et al., 2018). Mild cognitive impairment (MCI) is a transitional stage between normal cognitive functioning and dementia (Albert et al., 2011). Approximately 15% to 20% of people aged ≥ 65 years have MCI and are susceptible to dementia, with a higher conversion rate (Roberts and Knopman, 2013). AD is characterized by memory decline, which is related to pre-mature atrophy of the hippocampus, entorhinal cortex, and other medial temporal lobe structures (Hatashita and Yamasaki, 2013). Alteration in olfactory function often coincides with clinical symptoms and may even precede it (Hawkes, 2003). Olfactory dysfunction (OD) typically occurs in the prodromal stage of AD and can progress to the disease. Since early detection is crucial to prevent and slow progression, OD has been considered as a potential clinical marker for AD prediction, severity, and progression (Servello et al., 2015; Zou et al., 2016b).

Olfactory structures, such as the entorhinal cortex, amygdala, hippocampus, caudate, and other medial temporal lobes have been discovered (Kovács et al., 1999; Karas et al., 2003) to contain classic pathological features, such as neurofibrillary tangles and amyloid-β plaques, which are also observed in olfactory regions in early stage AD and MCI patients, including the olfactory bulb and tract and anterior olfactory nucleus (Hyman et al., 1991). Studies have suggested that aggregation of Aβ and tau proteins occurs in the olfactory neuroepithelium. Nevertheless, the central olfactory structures play a more important role in olfactory dysfunction. Impaired odor identification during lifetime was found to be robustly related to increased density of tangles in the entorhinal cortex and CA1/subiculum region of the hippocampus, but unrelated to other cortical sites after death (Wilson et al., 2007).

Hippocampal atrophy and volumetric measurements are included among the biomarkers of neuronal injury in MCI and AD diagnosis (Albert et al., 2011). In recent years, the link between olfactory identification performance and hippocampal atrophy has been recognized in some cross-sectional and longitudinal studies (Murphy et al., 2003; Kjelvik et al., 2014; Marigliano et al., 2014; Hagemeier et al., 2016). These positive results suggest that olfactory deficits may be a potential biomarker of hippocampal function. The aim of this systematic review and meta-analysis was to examine whether olfactory deficits correlate quantitatively with hippocampal atrophy, and to provide a comprehensive overview of the circumstances under which this correlation may be prominent due to different moderation factors.

## Method

### Search Strategy

Our meta-analysis was prepared according to the PRISMA guidelines and checklist (http://www.prisma-statement.org/PRISMAStatement/Checklist) and was registered with insplay.com. (Systematic Registration Number: INPLASY202140088; 10.37766/inplasy2021.4.0088) Two researchers (M-WS, S-SW) separately conducted an online search for papers from the PUBMED, EMBASE, and WEB OF SCIENCE databases from January 2003 to June 2020 using the MESH terms “Alzheimer's disease” and free words “olfactory” and “hippocampus OR hippocampal” (in the title/abstract). A complementary search of “Mild cognitive impairment” (free words in the title/abstract) substituting “Alzheimer's disease” was repeated. Among the results, we read through the abstract to include the studies that could potentially meet the criteria, then screened the full article for further verification, as well as relevant articles from the references in the full text for Supplementary Material.

### Study Selection

Studies were included if they met the following criteria: (1) participants with clinical diagnosis of amnestic AD or MCI were involved, with or without a health control; (2) both olfactory testing and hippocampal volumetric counting from MRI images were conducted from both hemisphere; (3) the correlation coefficient could be extracted directly or through calculation from the raw data; (4) studies in English published in peer-reviewed journals from 2003 onwards; (5) study type was a cohort study, case-control or cross-sectional study. The results were filtered to include only those written in English and conducted on living humans.

### Quality Assessment

The methodological quality of the included studies was assessed using the Newcastle-Ottawa Quality Assessment Scale (NOS) (Wells et al., 2013) by two independent researchers (M-WS and T-YC). Quality evaluation was applied to assess non-randomized studies. The NOS scale contains four domains including patient selection, comparability, and ascertainment of exposure or outcome of interest for case-control or cohort studies. The scale is assigned from 0 to 9 points, with studies scoring ≥ 7 points being considered high quality.

### Data Extraction

The coefficient *r* between olfactory test scores and hippocampal volume (either calculated using the Pearson or Spearman method) were extracted in eligible studies, which could be either in total (left and right hippocampal volume) or bilaterally (left or right hippocampal volume). In some studies, the *r*-values were tabulated directly. For others in which these values were absent, SPSS 22.0 software (IBM, Inc., Chicago) was used to calculate the Pearson correlation coefficient if the raw data was obtainable.

However, the *r*-value usually does not follow a normal distribution. Since the variance strongly depends on the correlation, it usually cannot be directly synthesized. The bias from these sample correlations could be partially eliminated through correction of the Fisher estimator (Berry and Mielke, 2000). Thus, an *r* to *Z* transformation—Fisher's *z* transformation—was introduced. The correlation was converted to Fisher's *z*-scale to obtain a normal distribution.

In each study, the effect size was transformed into *z* through the equation *z*' = 0.5 [ln (1 + *r*) – ln (1 – *r*)]. Then, the syntheses of *z* were performed in the meta-analysis.

### Statistical Analysis

Meta-analysis was conducted in *R* language with “meta” package in R-studio Version 1.3.959 (https://rstudio.com/), where random and fixed effect models were applied according to the heterogeneity test. The *I*^2^ statistic was calculated to assess the heterogeneity between studies. We attempted to fit a fixed effect model when the *I*^2^-value is <50%. An *I*^2^-value >50% or *p*-value < 0.05 suggests a rather heavy inconsistency and high heterogeneity, so we chose a sensitivity and subgroup analysis to render it and further discuss the potential sources.

Subgroups were divided into the following categories: (1) participants, patients/normal; (2) sides, left/right/both; and (3) age groups with a difference of 5 years.

## Results

### Description of Included Studies

**Figure 1:**
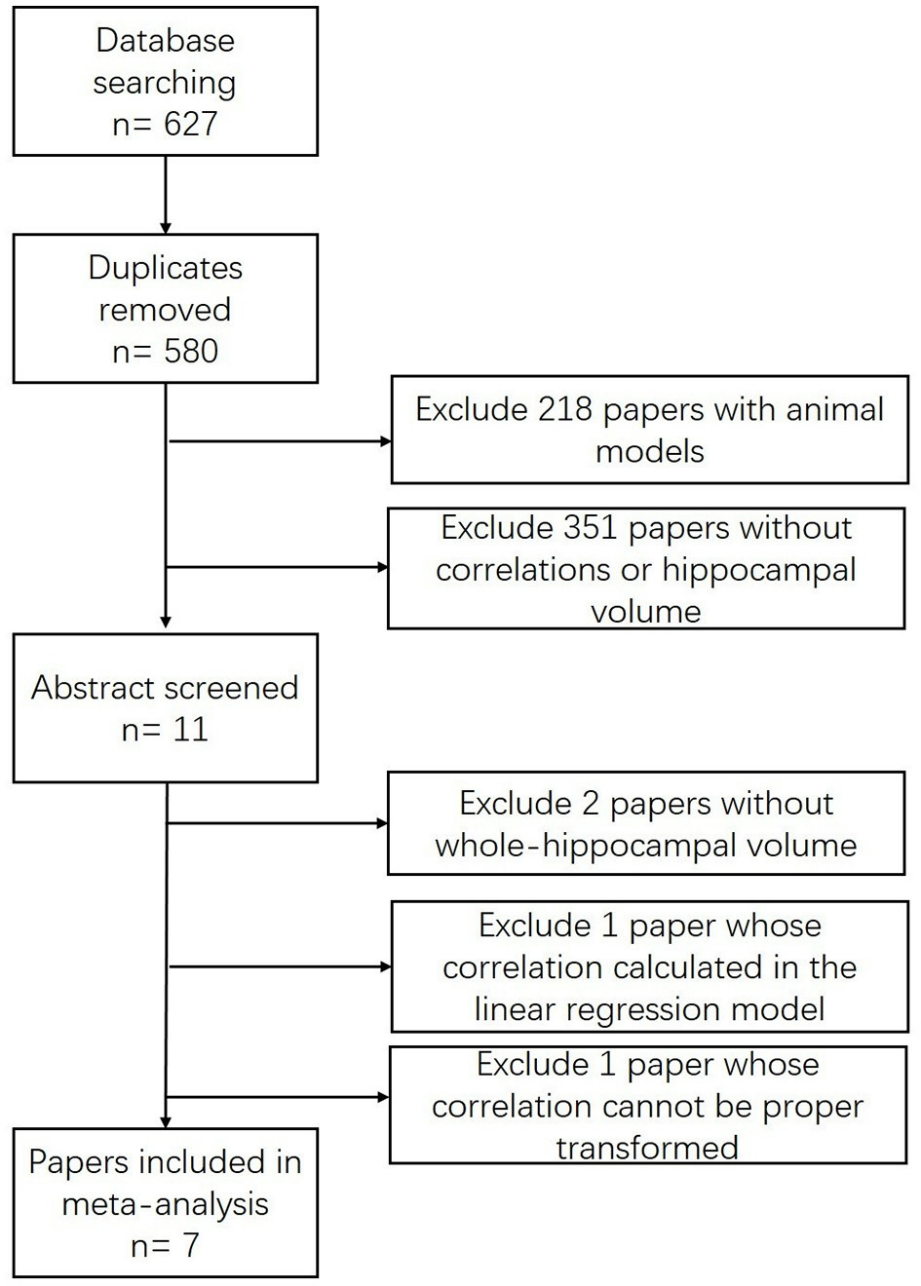
Flow chart of study selection.

Our search strategy initially identified 627 citations (Figure 1). After removing 47 duplicates, 575 studies were excluded by viewing the abstract for the animal model (*n* = 218) or non-relevance (*n* = 351). Eleven papers met the inclusion criteria (Murphy et al., 2003; Devanand et al., 2008, 2010; Wang et al., 2010; Lojkowska et al., 2011; Kjelvik et al., 2014; Marigliano et al., 2014; Vasavada et al., 2015; Hagemeier et al., 2016; Wu et al., 2019; Yu et al., 2019), among which four studies were excluded by screening the full article for specific reasons: the correlation in one study (Devanand et al., 2008) cannot be calculated or extracted through proper methods due to incomplete records; another (Kjelvik et al., 2014) presented a coefficient in a linear regression model; and two studies demonstrated the hippocampal volume either in an fMRI activated form (Wang et al., 2010) or volume changes in a 24-month follow-up study (Lojkowska et al., 2011).

A total of seven studies were included in the meta-analysis (Table 1). Five of the seven studies were considered high-quality (Table 2). Follow-up research was performed in a pilot study (Marigliano et al., 2014) which contains a baseline TDI score, hippocampal volume, and 12-month follow-up data. We computed the Pearson correlation coefficient *r* from the baseline data, since the baseline participants were all clinically confirmed aMCI participants. A cohort study (Devanand et al., 2010) initially enrolled 1,092 participants, 571 of whom had undergone hippocampal volume measurement with olfactory data.

**Table 1:** Demographic data and relevant parameters.

**References**	**Subject**	***N***	**Age**	**Sex (M/F)**	**Olfactory test**	**MMSE**	**Correlation** ***r***		**Side(s)**
Devanand et al., 2010	MCI	571	–	–	UPSIT	–	MCI + NC	0.16	Double
	NC								
Hagemeier et al., 2016	aMCI	19	73.6 ± 11	9/10	22.9 ± 8.6	–	AD	0.394	Right
	AD	42	76 ± 9	18/24	21.1 ± 7.9		aMCI	0.675	
	NC	19	69.4 ± 2.9	6/13	30.0 ± 6.7		NC	−0.185	
					UPSIT		AD	0.364	Left
							aMCI	0.438	
							NC	−0.132	
Vasavada et al., 2015	MCI	21	73.2 ± 9.0	10/11	24.2 ± 8.6	26.5 ± 1.9	AD + MCI + N	0.55	Double
	AD	16	71.9 ± 11.9	5/10	15.5 ± 8.4	18.9 ± 5.4	AD + MCI	0.33	
	NC	27	69.5 ± 10.4	12/15	34.0 ± 4.2	28.5 ± 1.5			
					UPSIT				
Wu et al., 2019	MCI	27	68.04 ± 7.58	13/14	CSIT	26 (25, 28)	AD	0.242	Right
	AD	37	66.86 ± 10.27	17/20		16.03 ± 4.04	MCI	0.231	
	NC	30	67.23 ± 6.71	11/19		29 (28, 30)	NC	0.167	
							TOTAL	0.512	
							AD	0.323	Left
							MCI	0.088	
							NC	0.326	
							TOTAL	0.512	
Yu et al., 2019	MCI	31	65.9 ± 7.9	14/17	UPSIT	–	MCI + NC	0.42	Right
	NC	9	66.44 ± 7.05	3/6			MCI + NC	0.55	Left
Marigliano et al., 2014	aMCI	18	68.05 ± 3.5	9/9	SSET	–		0.508	Double
Murphy et al., 2003	AD	13	73.08 ± 2.19	8/5	SDOIT	22.85 ± 1.04	AD	0.54	Right
	NC	22	72.45 ± 1.78	10/12		29.68 ± 0.12	NC	0.23	
							AD	0.85	Left
							NC	0.17	

*M/F, Male/Female; MMSE, Mini-Mental State Examination; UPSIT, University of Pennsylvania Smell Identification Test; CSIT, Chinese smell identification test; SSET, Sniffin Sticks Extended Test; SDOIT, San Diego Odor Identification Test; MCI, mild cognitive impairment; NC, normal control; AD, Alzheimer's disease*.

**Table 2:** The Newcastle-Ottawa scale (NOS).

**References**	**Selection**				**Comparability**	**Exposure**			**Scores**
	**Adequate definition of cases**	**Representativeness of the cases**	**Selection of controls**	**Definition of controls**	**Control for important factor**	**Ascertainment of exposure**	**Same method of ascertainment for cases and controls**	**Non-response rate**	
Hagemeier et al., 2016	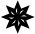	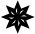	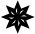	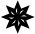	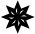	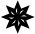	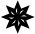	–	7
Vasavada et al., 2015	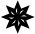	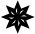	–	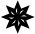	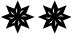	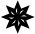	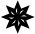	–	7
Wu et al., 2019	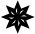	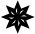	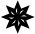	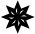	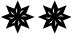	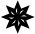	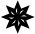	–	8
Yu et al., 2019	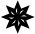	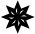	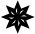	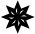	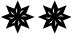	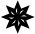	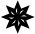	–	8
Murphy et al., 2003	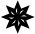	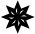	–	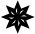	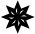	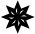	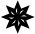	–	6
	**Selection**				**Comparability**	**Outcome**			
	**Representativeness of the exposed cohort**	**Selection of the non-exposed cohort**	**Ascertainment of exposure**	**Demonstration that outcome of interest was not present at start of study**	**Comparability of cohorts on the basis of the design or analysis**	**Assessment of outcome**	**Enough follow-up of cohorts**	**Adequacy of follow-up of cohorts**	
Devanand et al., 2010	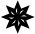	–	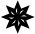	–	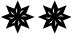	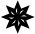	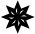	–	6
Marigliano et al., 2014	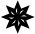	–	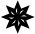	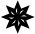	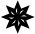	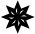	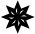	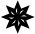	7

*A study can be awarded a maximum of one star for each numbered item within the selection and exposure categories. A maximum of two stars can be given for comparability*.

All seven studies yielded 22 effect sizes and 902 participants. The participants were clinically diagnosed with MCI/AD or normal controls. In four studies (Murphy et al., 2003; Hagemeier et al., 2016; Wu et al., 2019; Yu et al., 2019), the correlation coefficients were computed bilaterally according to hippocampal volume measurements on each side. In the three remaining studies (Devanand et al., 2010; Marigliano et al., 2014; Vasavada et al., 2015), *r* was calculated from the double-sided volume in total.

### Association Between Olfactory Tests Score and Hippocampal Volumes

**Figure 2:**
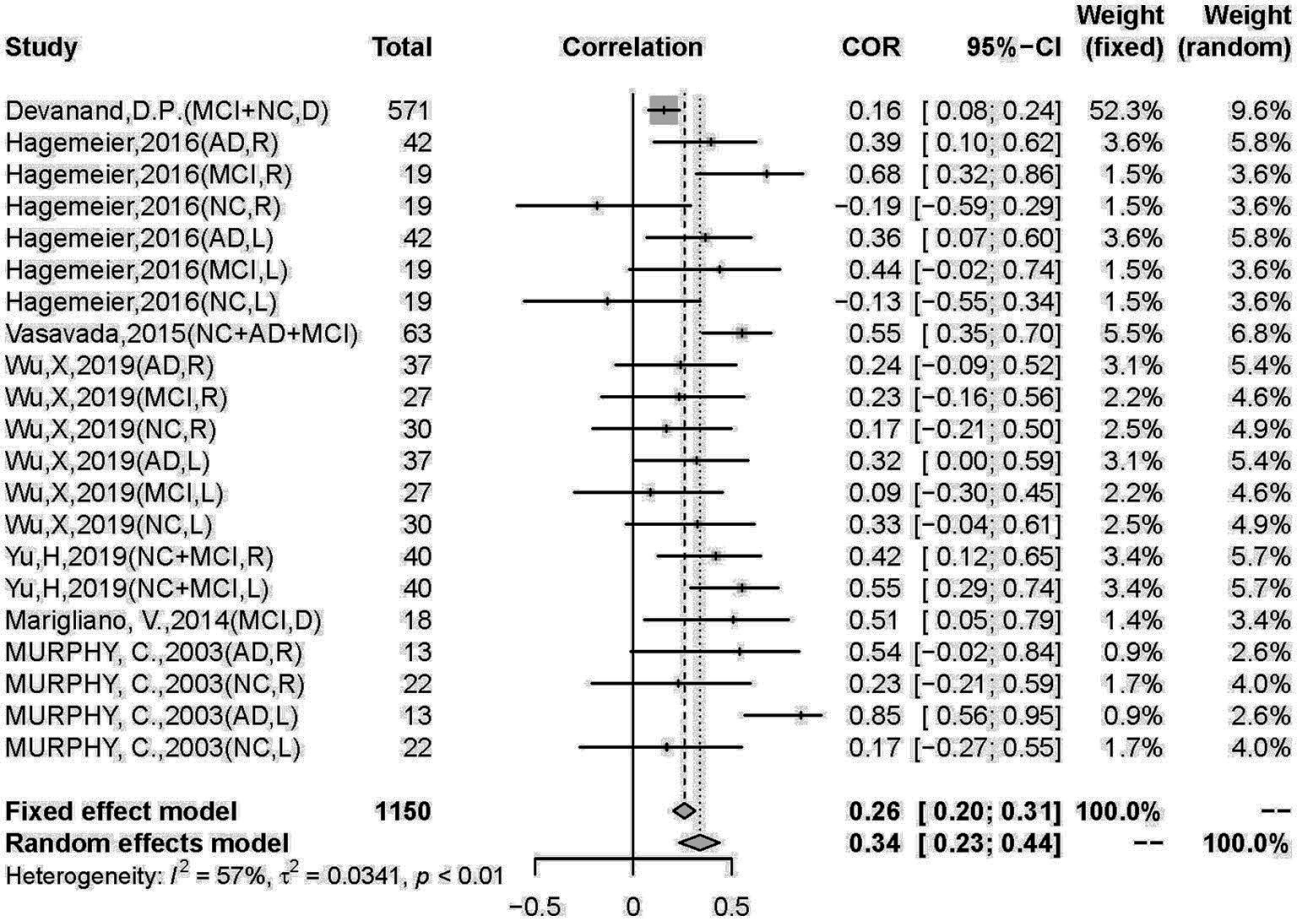
Forest plot summarizing the overall correlation between odor identification score and hippocampal volume across all studies and their 95% interval for each study. (Random effects model selected. NC, normal control; AD, Alzheimer's disease; MCI, mild cognitive impairment; D, hippocampal volume measurement in double sides; R, hippocampal volume measurement in right side; L, hippocampal volume measurement in left side).

**Figure 3:**
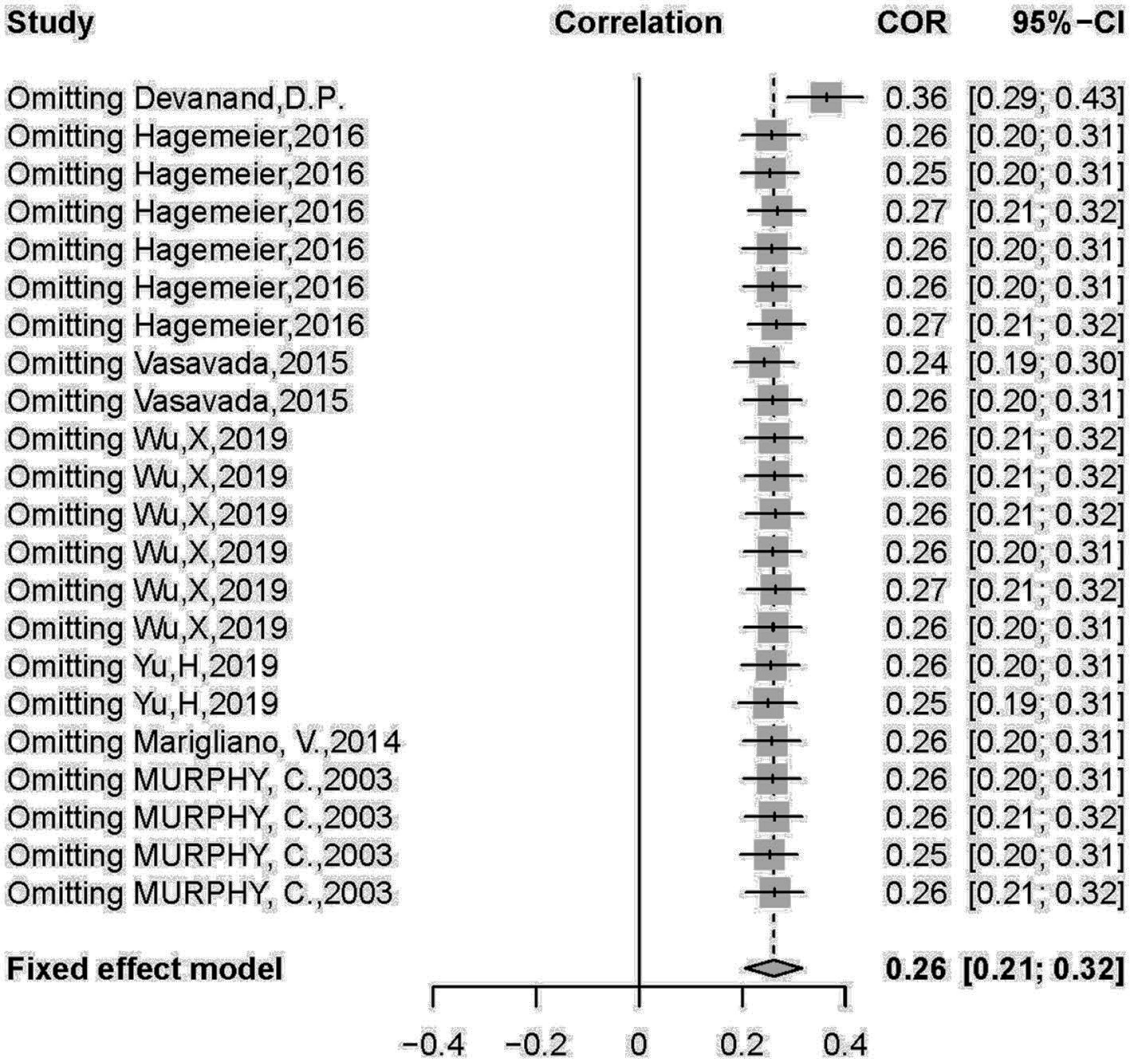
Sensitive analysis in leave-one-out method.

There was a positive correlation between olfactory test scores and hippocampal volume (*r* = 0.3392, 95% CI: 0.2335–0.4370, *p* < 0.0001) (Figure 2). Egger's regression test revealed an overall reporting bias (*p* = 0.029). A trim-and-fill funnel plot showed a weak positive correlation (*r* = 0.2074, 95% CI: 0.0876–0.3214, *p* < 0.0001). Further, an influential analysis identified that no outliers in the included studies could reverse the analytical results using the leave-one-out method (Figure 3). Moreover, there was moderate heterogeneity in the sample of all included studies (*I*^2^ = 57%, *p* < 0.01).

### Moderator Effects

To investigate potential sources of heterogeneity, we performed a subgroup analysis with several moderator variables, including patient type, age, hemisphere, and olfactory tests. The following results revealed that patient type and age might be the two possible sources of heterogeneity.

**Figure 4:**
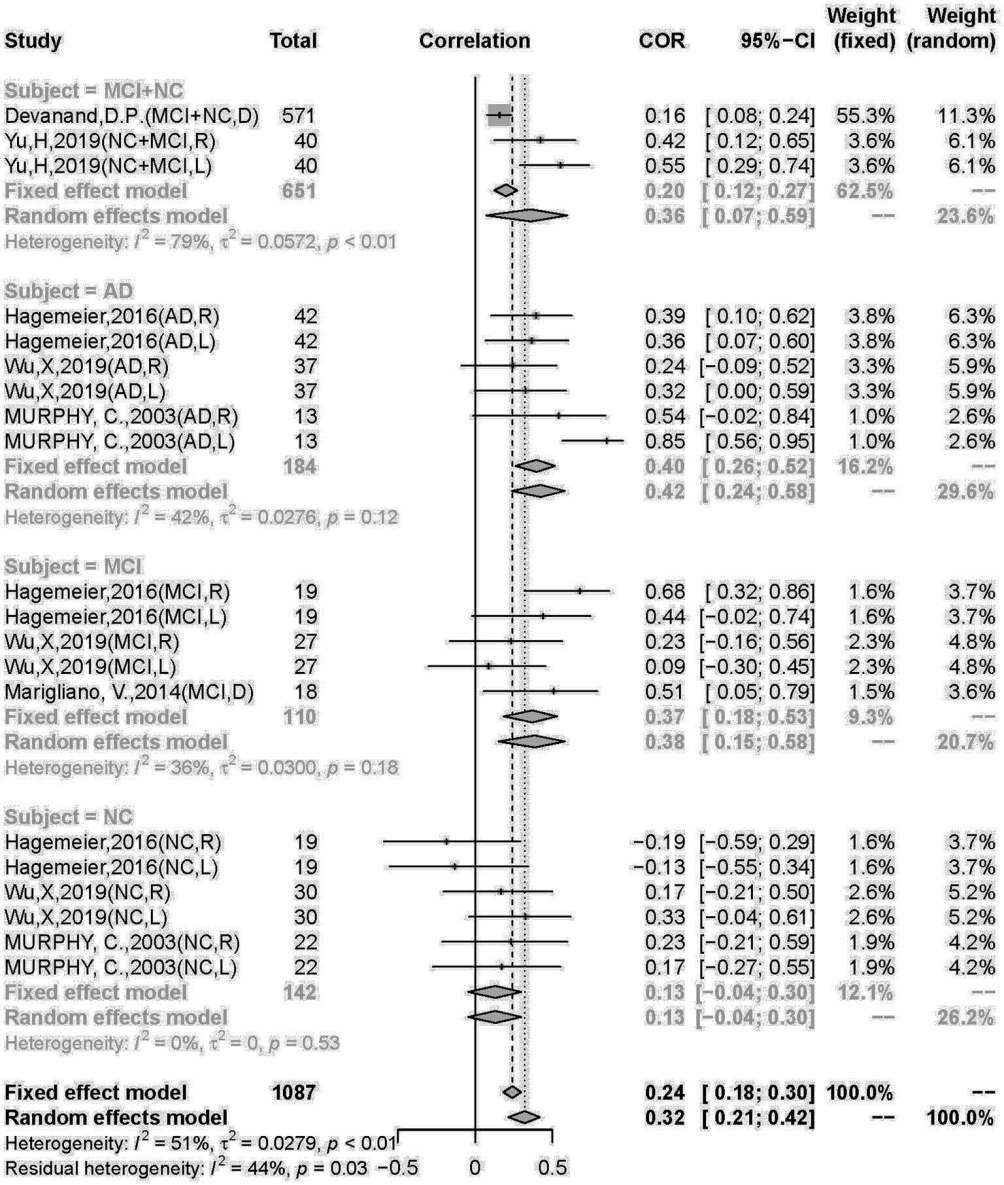
Subgroup analysis in different subject in group of AD, MCI, NC.

A significant difference in the correlation between the AD, MCI, and NC groups was discovered. The moderator analysis for patient type was significant (*Q* = 17.64; *p* = 0.0014), suggesting that this variable may contribute to heterogeneity. Subgroups of AD (*r* = 0.3959, 95% CI: 0.2605–0.5160, *k* = 6), MCI (*r* = 0.3691, 95% CI: 0.1841–0.5288, *k* = 5), and NC (*r* = 0.1305, 95% CI: −0.0447–0.2980, *k* = 6) were not significant in heterogeneity (AD: *I*^2^ = 27%, *p* = 0.12; MCI: *I*^2^ = 36%, *p* = 0.18; *I*^2^ = 0%, *p* = 0.53). The differences were not significant between AD and non-AD (MCI + NC) (AD: *r* = 0.4222, 95% CI: 0.2372–0.5776, *k* = 6; non-AD: *r* = 0.2728, 95% CI: 0.1494–0.3879, *k* = 14; *p* = 0.1735), AD and MCI (*p* = 0.8072), but significant in AD and NC (*p* = 0.0154) and AD. The correlation was significantly stronger in the patient group than in the control group (*p* = 0.0121) and in the AD group than in the MCI group, indicating a pathology-dependent penetrance (Figure 4).

The olfactory deficits were found to be most correlated in the age range of 70.6–75.6 years old (*r* = 0.5113, 95% CI: 0.3181–0.6637, *k* = 7) showing a low risk of heterogeneity (*I*^2^ = 46%, *p* = 0.08), and more predominantly than the 65.6–70.6 years group (*r* = 0.2698, 95%CI: 0.1376–0.3926, *k* = 11) and the 75.6–80.6 years group (*r* = 0.2591, 95% CI: 0.0809–0.4211, *k* = 3). The mean age of all the participants was 75.20 years (range from 66.86 to 80.6 years). For a mean age difference of 5 years, the moderator analysis was statistically significant (*Q* = 17.14, *p* = 0.0002).

The moderator analysis for hemisphere was not significant (*Q* = 5.02, *p* = 0.0811), suggesting that lateralization of odor memory might not contribute to the observed heterogeneity. Moreover, no obvious hemispheric dominance was found in olfaction (left: *r* = 0.35, 95% CI: 0.2318–0.4615, *I*^2^ = 53%, *p* = 0.03; right: *r* = 0.31, 95% CI: 0.1905–0.4268, *I*^2^ = 27%, *p* = 0.20). We further investigated the lateralization among patient groups and subgroup effects in the left hippocampus group. The hemispheric parameters in patients were not significant.

In all seven studies, odor identification scores were obtained using various methods: the University of Pennsylvania Smell Identification Test (UPSIT) in four studies (Vasavada et al., 2015; Hagemeier et al., 2016; Marin et al., 2018; Yu et al., 2019), the Chinese smell identification test (CIST) in Wu et al. (2019), the Sniffin Sticks Extended Test (SSET) in Marigliano et al. (2014), and the San Diego Odor Identification Test (SDOIT) in Murphy et al. (2003). The subgroup analysis revealed that the difference between the types of olfactory identification tests was not significant (*Q* = 3, *p* = 0.3916).

Given the lack of demographic figures for gender information, the pooled *r*-value categorized by sex was unable to be detected. The subgroup analysis revealed that part of the heterogeneity was due to subject type and age.

## Discussion

Our meta-analysis explored the relationship between odor identification decline and hippocampal atrophy in AD and MCI patients with normal controls. The main result obtained from our meta-analysis showed a significant positive correlation (*r* = 0.3392, 95% CI: 0.2335–0.4370, *p* < 0.0001) between olfactory identification deficits and hippocampal atrophy. A prominent difference was noted in the MCI/AD group, with a stronger correlation than the control group (*p* = 0.0121). In addition, the association in the AD group was stronger than in the MCI group, suggesting that odor identification decline could be detected early in the MCI stage and followed the disease progression.

Moderate heterogeneity was detected, suggesting that the overall combination of associations might not be present across all contexts. This may be due to clinical heterogeneity in the variation in participants, and the diversity of participant numbers could considerably affect the precision of the statistical results. The moderator analysis showing patient types and age were the two main variables that might be most likely to account for heterogeneity. In addition, half of the sample size was due to one study alone whose *r*-value was nearly negligible (*r* = 0.157), but stronger relationships tended to be observed in smaller samples. Although no outliers were identified, the study of Devanand et al. (2010) has influenced the overall effect size to a greater extent for those with a heavier weight. Typically, sample sizes are reciprocal to the precision of the estimated effects (Sedgwick and Marston, 2015), and studies with larger sample sizes are given for more weight in analysis. Therefore, sample size is considered to affect heterogeneity, and thus studies with larger sample sizes are necessary for further validation. Additional unpublished papers and non-English results should also be involved to further reduce heterogeneity.

Patient type was an independent factor in OD. Olfactory identification deficits were more prominently correlated with hippocampal atrophy in the AD group than in the MCI group, both of which were consistently stronger than in the normal control group. Previous meta-analyses have validated similar results. Rahayel et al. (2012) conducted a meta-analysis and confirmed that AD has severe detrimental effects on olfactory function across the whole spectrum, but has a stronger effect on odor identification than odor detection. Olfactory identification was the most impaired among all domains in MCI (Roalf et al., 2017) and AD patients. Kotecha et al. (2018) systematically reviewed and concluded that olfaction progressively worsens from MCI to AD, which highlights the potential utility of olfactory identification tests as prognostic tools for AD (Sun et al., 2012). Jung et al. (2019) reported similar results, revealing that olfactory identification was more profoundly impaired in AD than in MCI; further, Roalf et al. (2017) concluded a more extensively impaired odor identification in MCI. The former result is compatible with our finding that the relationship in AD is higher than in MCI groups (MCI: *r* = 0.3691; AD: *r* = 0.3959; *p* = 0.081). This clear increase in odor identification deficits from cognitively normal to MCI and AD has been described in both clinical and epidemiological studies (Graves et al., 1999; Schubert et al., 2008; Devanand et al., 2015). In addition, this increase in correlation with disease progression might indicate that the olfactory cortex (hippocampus as the second olfactory cortex) is compromised through the pathophysiological continuum (Bathini et al., 2019) of sequential events of the pathology of the disease.

It is widely accepted that odor identification generally declines with normal aging, especially over age 70 (Doty et al., 1984). Significant age-related alterations have been observed in odor identification tests in various studies. In functional magnetic resonance imaging (fMRI), there is a decrease in the activation of olfactory-related regions in the elderly (Suzuki et al., 2001; Ferdon and Murphy, 2003). This was in line with a longitudinal study showing an inverse correlation of B-SIT scores before death and post-mortem density of neurofibrillary tangles in the entorhinal cortex, the CA1 subfield of the hippocampus. Our pooled correlation in age was predominant in patients between the ages of 70–75, showing a moderate association (*r* = 0.5113, 95% CI: 0.3181–0.6637). This result did not explain the progressive trend in olfactory impairment. Thus, we speculate that this is due to the discontinuity of the wide age interval. We re-analyzed a 2-year interval in patient and control groups separately, and discovered that the growth of correlation increases with age (66–68: *r* = 0.2953, 95% CI: 0.1030–0.4664; 70–72: *r* = 0.2521, 95% CI: 0.0060–0.4694; 72–74: *r* = 0.4554, 95% CI: 0.2434–0.6259; 74–76: *r* = 0.4679, 95% CI: 0.2999–0.6078; *Q* = 15.18, *p* = 0.2317). This indicates that aging could be an independent factor for odor identification deficits when the magnitude of the disease was ruled out. Thus, we inferred that age-dependent hippocampal volume decrement clouds affect olfactory function physiologically; on the other hand, this physiological function could be worsened under the pathological extension from MCI to AD.

Previous studies have suggested that odor memory is lateralized to the right hemisphere (Jones-Gotman and Zatorre, 1993; Olsson and Cain, 2003). The right hippocampus was found to be larger in the NC and MCI groups, while there was no significant difference in AD in Wolf et al.'s (2001) study. Zou et al. (2016a) concluded that the right hemisphere is predominant in odor hedonic judgment. In contrast, fMRI brain scans of brain activation are generally lateralized to the left hemisphere when received pleasant smell of odors, and unpleasant smells to the right (Henkin and Levy, 2001). However, the controversial hemispheric prominence generally did not include the hippocampus. Our analysis indicated that there were no significant hemispheric differences. One study (Murphy et al., 2003) reported a stronger correlation in the left hippocampus over the right (*r* = 0.85, *p* < 0.001), which made our heterogeneity in the hemispheric moderator on the left side significant. We would assume that the current, small numbered, and conflicting results require further observation.

It can be affirmed that our results in brain-behavior relationships are congruent with previous meta-analyses that have validated olfactory dysfunction in AD. However, the correlation between hippocampal atrophy and odor identification deficits is by far the first to be explored, which could be a key explanation for the hypothesis that it is generated from the pathology burden in the medial-temporal lobe. Consequently, olfactory deficits originate in central structures, suggesting that odor identification and recognition tests could be beneficial for the early detection of subclinical cases.

Several clinical studies have observed that OD and cognitive impairment share the same anatomical modifications of AD-signature cortex decrease (Lian et al., 2019), especially the olfactory cortex and the hippocampus (Al-Otaibi et al., 2020). In recent years, a link between olfactory deficits and AD has been consistently reported. It is commonly recognized that prior to cognitive symptoms (Price et al., 1991; Jellinger and Attems, 2005; Attems and Jellinger, 2006), AD pathology appears in the trans-entorhinal region, entorhinal cortex, hippocampus and successively in olfactory bulb (OB), olfactory tract, and other structures (Ohm and Braak, 1988; Kovács et al., 1999). However, the mechanisms underlying the relationship between odor identification (OI) and hippocampal pathology have not been fully elucidated. Evidence suggests that neuroinflammation occurs in Aβ burden structures (Hanzel et al., 2014). A decrease in hippocampal volume is associated with hippocampal-dependent dysfunction in learning and memory (Ziehn et al., 2010), which also correlates with microglial activation, synaptopathy/synaptic loss, and neurodegeneration (Mandolesi et al., 2010; Girard et al., 2014). Soluble Aβ accumulation in the OB is strongly correlated with early olfactory dysfunction in both AD patients and mouse models (Wesson et al., 2010). Further, a recently published meta-analysis by Tu et al. (2020) discovered a weak negative correlation between OI ability and cerebral Aβ PET (*r* = −0.25, *P* = 0.008) and CSF tau (*r* = −0.17, *p* = 0.006) levels. The specificity was speculated to be the marginal burden of pathological changes that implicate OI ability. The review concluded that the combination of OI tests and other biologic markers still preserves the predictive value of assessing cognitive decline and progression from MCI to AD. However, this may conversely explain the hypothesis that soluble toxic aggregates of both Aβ and tau can self-propagate and spread throughout the brain by prion-like mechanisms (Goedert et al., 2010; Bloom, 2014), and propagation of proteotoxicity along the olfactory nerve could likely affect olfactory-ERC-hippocampal circuits (Busche et al., 2008; Rey et al., 2018). Oligomeropathy (Forloni and Balducci, 2018), neuroinflammation, and the prion-like hypothesis may trigger olfactory dysfunction.

Our study has several limitations. First, there is inadequate inclusion of studies aiming at olfactory discrimination and detection threshold, along with studies reporting a correlation between OB and olfactory epithelium deficits and hippocampal atrophy. Odor discrimination and detection thresholds (Mesholam et al., 1998) were not adequately covered in our analysis. Second, according to the subgroup analysis, we could confirm that aging is one of the moderator factors; however, the linear regression could not be drawn from the present discontinuous data. Furthermore, heterogeneity in sample size preserves obvious differences in the statistical results, which could affect precision. Thus, meticulously designed studies with larger sample sizes are necessary for validation.

## Conclusion

This meta-analysis quantified a positive correlation between olfactory identification deficits and hippocampal atrophy. The correlation appears to be more predominant in MCI and AD patients, suggesting that olfactory identification deficits appear in the early stages of the continuum. Age is an independent factor that affects the severity of the correlation during disease progression. The mildness of correlation suggests that olfactory tests may be more accurate in early detection when combined with other non-invasive examinations in AD.

## Data Availability Statement

The original contributions presented in the study are included in the article/Supplementary Material, further inquiries can be directed to the corresponding authors.

## Author Contributions

M-WS and J-NN wrote the manuscript. M-WS and T-YC performed the analysis. S-SW and M-WS helped to proofread the literature search. JS and J-ZT supervised the study. All authors contributed to the article and approved the submitted version.

## Funding

This work was supported by the National Natural Science Foundation of China (Grant No. 82074362).

## Conflict of Interest

The authors declare that the research was conducted in the absence of any commercial or financial relationships that could be construed as a potential conflict of interest.

## Publisher's Note

All claims expressed in this article are solely those of the authors and do not necessarily represent those of their affiliated organizations, or those of the publisher, the editors and the reviewers. Any product that may be evaluated in this article, or claim that may be made by its manufacturer, is not guaranteed or endorsed by the publisher.
