# Supplementary material for: The Correlation Between Olfactory Test and Hippocampal Volume in Alzheimer's Disease and Mild Cognitive Impairment Patients: A Meta-Analysis
Source: Front Aging Neurosci. 2021 Oct 20;13:755160. doi: 10.3389/fnagi.2021.755160 (PMC8564359; doi:10.3389/fnagi.2021.755160)
Supplement: Supplementary file 1 [file Data_Sheet_1.PDF]

Supplementary Material

1 Supplementary Figures

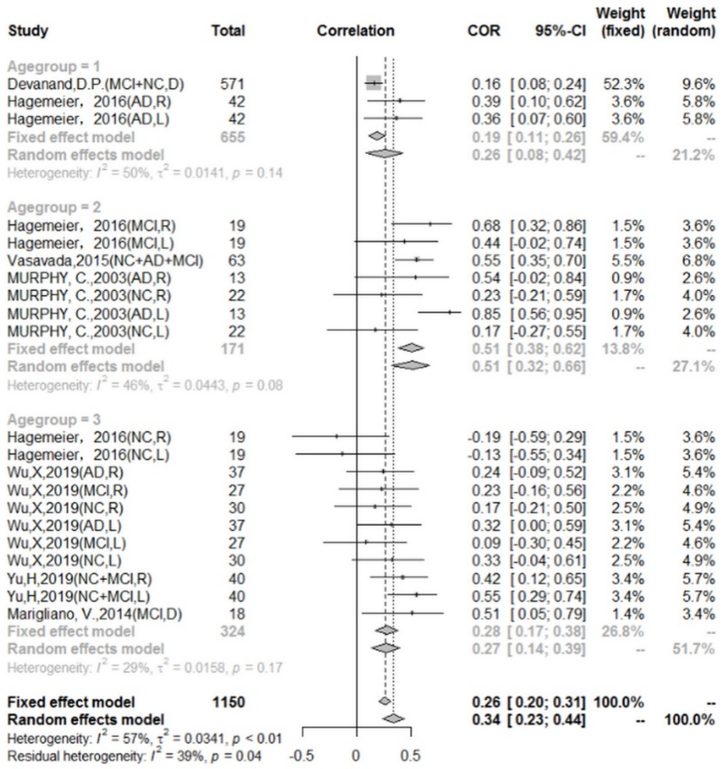

Supplementary Figure 1. Subgroup analysis of different age group. (Agegroup1:65.6-70.6 years; Agegroup2:70.6-75.6years; Agegroup3:75.6-80.6 years)

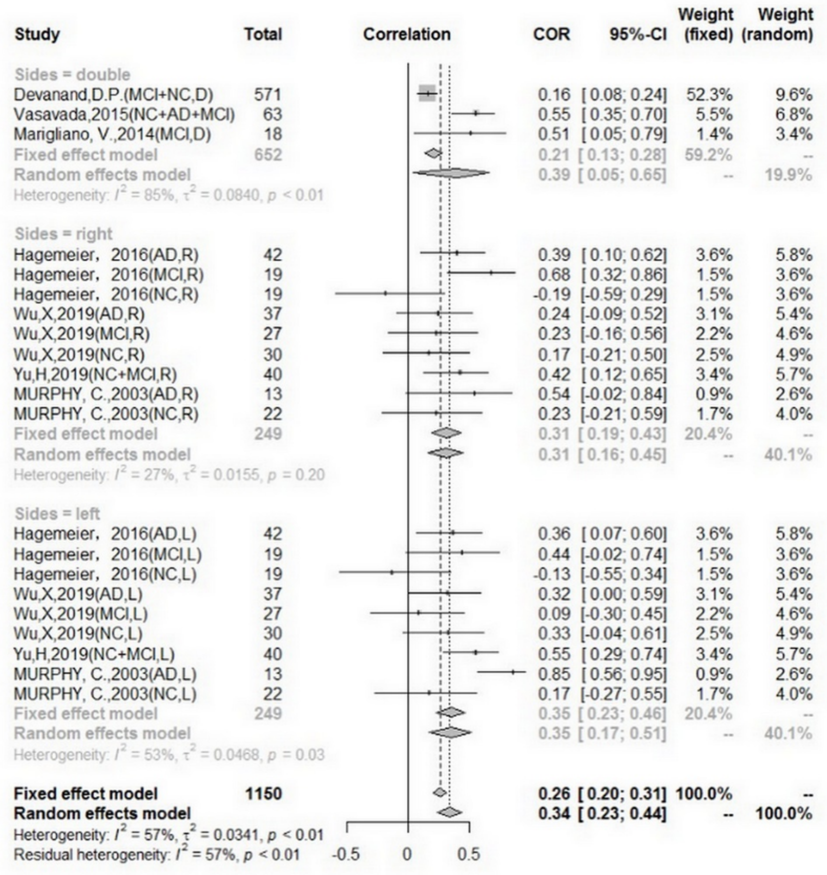

Supplementary Figure 2. Subgroup analysis of hippocampal volume in different hemisphere.
