# Supplementary material for: The Correlation Between Olfactory Test and Hippocampal Volume in Alzheimer's Disease and Mild Cognitive Impairment Patients: A Meta-Analysis
Source: Front Aging Neurosci. 2021 Oct 20;13:755160. doi: 10.3389/fnagi.2021.755160 (PMC8564359; doi:10.3389/fnagi.2021.755160)
Supplement: Supplementary file 2 [file Data_Sheet_2.PDF]

Supplementary Material

1 Supplementary Figures

Supplementary Figure 1.

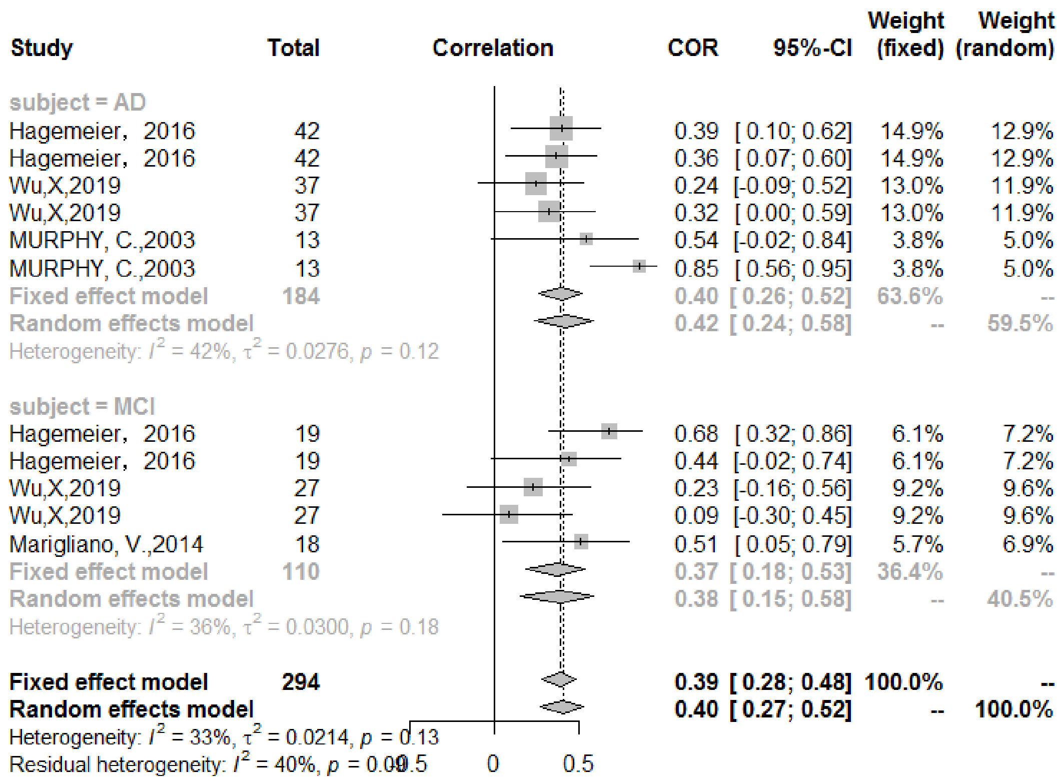

Supplementary Figure 2.

```
Number of studies combined: k = 11

                        COR           95%-CI    z  p-value
Fixed effect model   0.3862 [0.2784; 0.4843] 6.58 < 0.0001
Random effects model 0.4030 [0.2681; 0.5224] 5.49 < 0.0001

Quantifying heterogeneity:
 tau^2 = 0.0214 [0.0000; 0.2119]; tau = 0.1461 [0.0000; 0.4603];
 I^2 = 33.2% [0.0%; 67.2%]; H = 1.22 [1.00; 1.75]

Quantifying residual heterogeneity:
 I^2 = 39.6% [0.0%; 71.2%]; H = 1.29 [1.00; 1.86]

Test of heterogeneity:
     Q d.f. p-value
 14.96   10  0.1334

Results for subgroups (fixed effect model):
                k    COR           95%-CI    Q   I^2
subject = AD    6 0.3959 [0.2605; 0.5160] 8.65 42.2%
subject = MCI   5 0.3691 [0.1841; 0.5288] 6.25 36.0%

Test for subgroup differences (fixed effect model):
                  Q d.f. p-value
Between groups  0.06    1  0.8072
Within groups  14.90    9  0.0936

Results for subgroups (random effects model):
                k    COR           95%-CI  tau^2    tau
subject = AD    6 0.4222 [0.2372; 0.5776] 0.0276 0.1660
subject = MCI   5 0.3842 [0.1506; 0.5772] 0.0300 0.1731

Test for subgroup differences (random effects model):
                   Q d.f. p-value
Between groups   0.07    1  0.7866

Details on meta-analytical method:
- Inverse variance method
- DerSimonian-Laird estimator for tau^2
- Jackson method for confidence interval of tau^2 and tau
- Fisher's z transformation of correlations
```

Supplementary Figure 1&2. Forest plot for subgroup analysis of AD and MCI. (AD: r=0.3959, 95% CI: 0.2605 to 0.5160, k=6; MCI: r=0.3691, 95% CI: 0.1841 to 0.5288, k=5; p=0.8072)

Supplementary Figure 3.

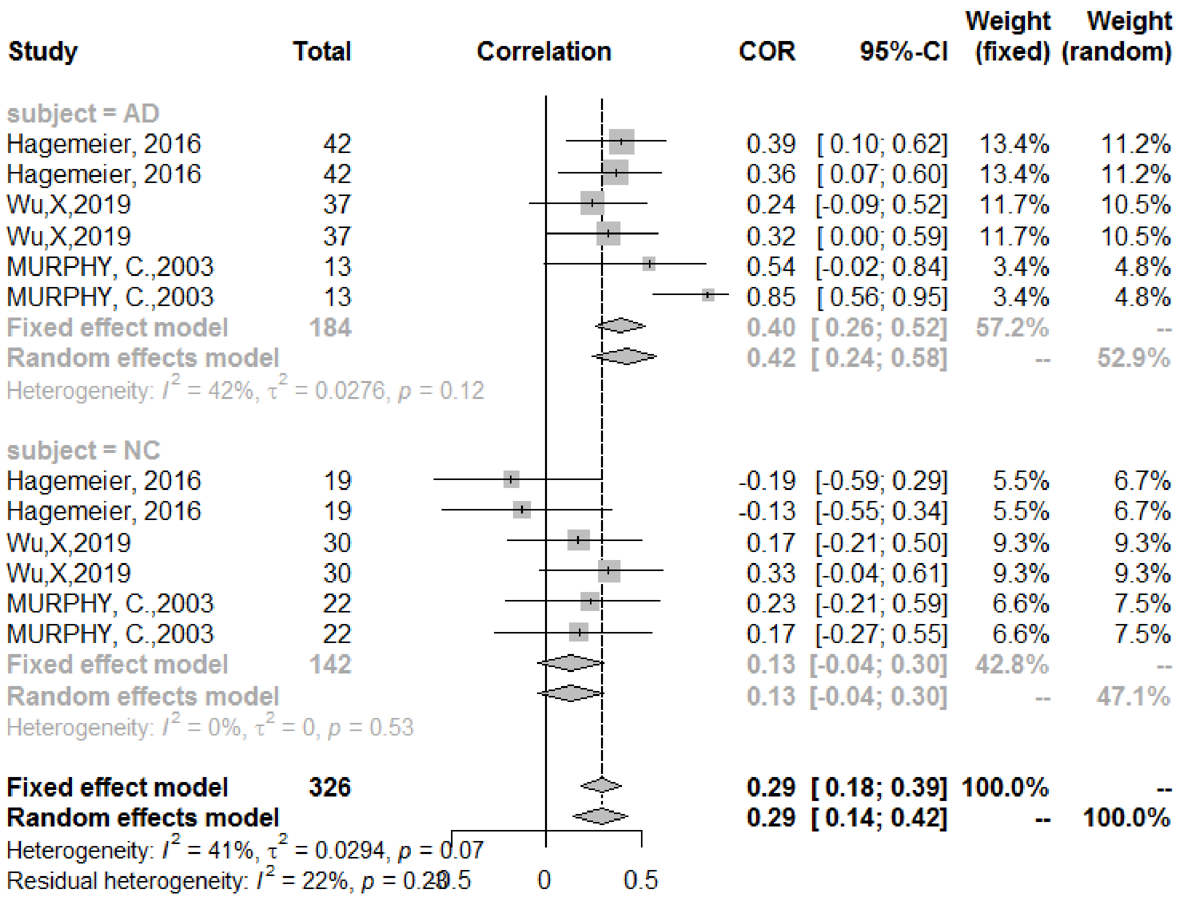

Supplementary Figure 4

```
Number of studies combined: k = 12

                        COR            95%-CI    z  p-value
Fixed effect model   0.2875 [0.1788; 0.3893] 5.04 < 0.0001
Random effects model 0.2871 [0.1405; 0.4213] 3.76   0.0002

Quantifying heterogeneity:
 tau^2 = 0.0294 [0.0029; 0.2422]; tau = 0.1714 [0.0538; 0.4921];
 I^2 = 41.1% [0.0%; 70.1%]; H = 1.30 [1.00; 1.83]

Quantifying residual heterogeneity:
 I^2 = 22.0% [0.0%; 60.9%]; H = 1.13 [1.00; 1.60]

Test of heterogeneity:
     Q d.f. p-value
 18.68   11  0.0670

Results for subgroups (fixed effect model):
               k    COR            95%-CI    Q   I^2
subject = AD   6 0.3959 [ 0.2605; 0.5160] 8.65 42.2%
subject = NC   6 0.1305 [-0.0447; 0.2980] 4.17  0.0%

Test for subgroup differences (fixed effect model):
                   Q d.f. p-value
Between groups  5.86    1  0.0154
Within groups  12.82   10  0.2340

Results for subgroups (random effects model):
               k    COR            95%-CI  tau^2    tau
subject = AD   6 0.4222 [ 0.2372; 0.5776] 0.0276 0.1660
subject = NC   6 0.1305 [-0.0447; 0.2980]      0      0

Test for subgroup differences (random effects model):
                    Q d.f. p-value
Between groups   5.25    1  0.0219

Details on meta-analytical method:
- Inverse variance method
- DerSimonian-Laird estimator for tau^2
- Jackson method for confidence interval of tau^2 and tau
- Fisher's z transformation of correlations
```

Supplementary Figure 3&4. Forest plot for subgroup analysis of AD and NC. (AD: r=0.3959, 95% CI: 0.2605 to 0.5160, k=6; NC: r=0.1305, 95% CI: -0.0447 to 0.2980, k=6; p=0.0154)

Supplementary Figure 5

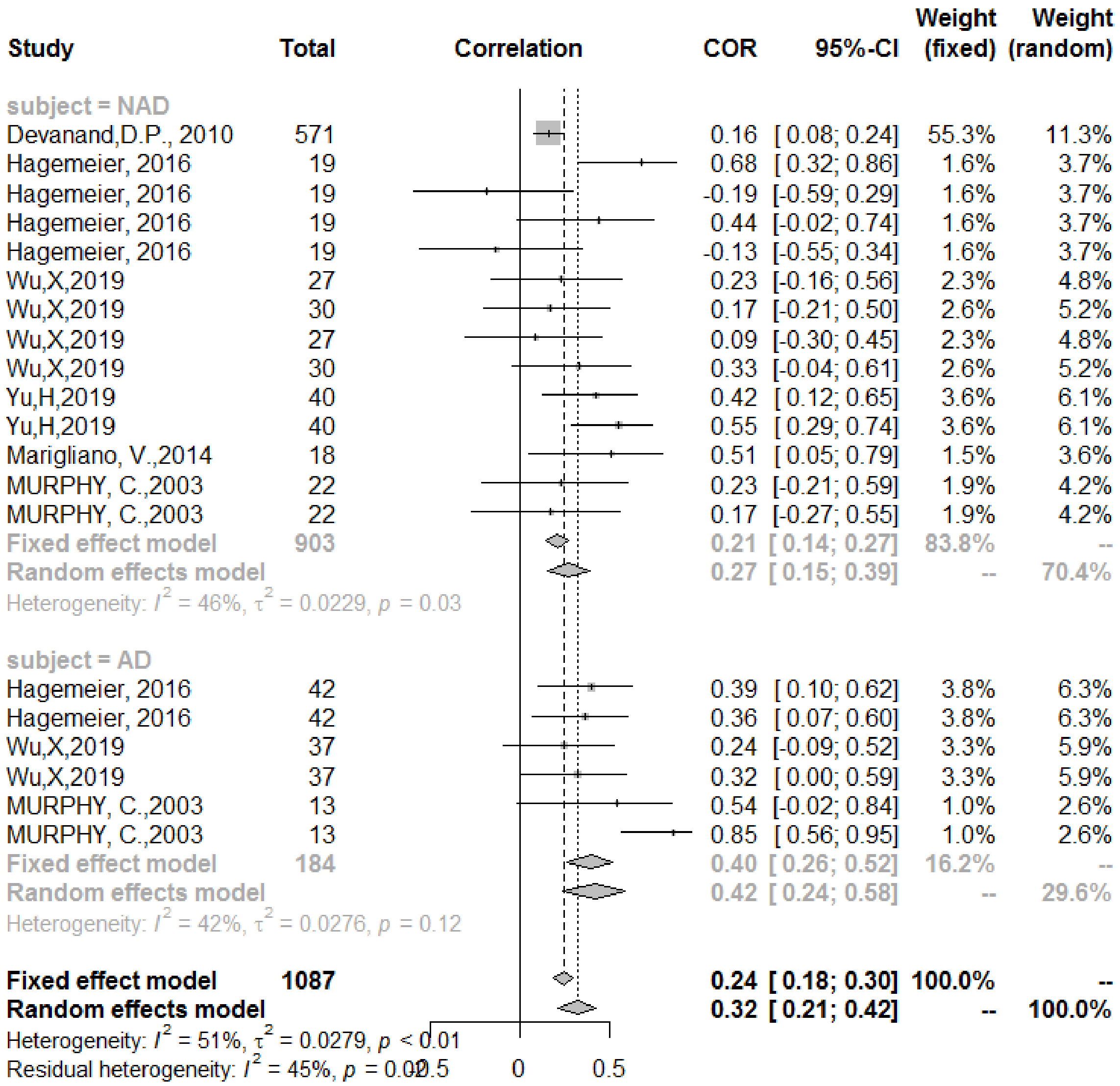

Supplementary Figure 6

```
Number of studies combined: k = 20

                        COR           95%-CI    z  p-value
Fixed effect model   0.2405 [0.1821; 0.2972] 7.86 < 0.0001
Random effects model 0.3198 [0.2143; 0.4179] 5.71 < 0.0001

Quantifying heterogeneity:
 tau^2 = 0.0279 [0.0061; 0.1313]; tau = 0.1671 [0.0781; 0.3623];
 I^2 = 50.7% [17.6%; 70.5%]; H = 1.42 [1.10; 1.84]

Quantifying residual heterogeneity:
 I^2 = 44.8% [5.3%; 67.8%]; H = 1.35 [1.03; 1.76]

Test of heterogeneity:
     Q d.f. p-value
 38.56   19  0.0050

Results for subgroups (fixed effect model):
                k    COR           95%-CI     Q   I^2
subject = NAD  14 0.2088 [0.1441; 0.2717] 23.95 45.7%
subject = AD    6 0.3959 [0.2605; 0.5160]  8.65 42.2%

Test for subgroup differences (fixed effect model):
                   Q d.f. p-value
Between groups  5.95    1  0.0147
Within groups  32.60   18  0.0186

Results for subgroups (random effects model):
                k    COR           95%-CI  tau^2    tau
subject = NAD  14 0.2728 [0.1494; 0.3879] 0.0229 0.1514
subject = AD    6 0.4222 [0.2372; 0.5776] 0.0276 0.1660

Test for subgroup differences (random effects model):
                    Q d.f. p-value
Between groups   1.85    1  0.1735

Details on meta-analytical method:
- Inverse variance method
- DerSimonian-Laird estimator for tau^2
- Jackson method for confidence interval of tau^2 and tau
- Fisher's z transformation of correlations
```

Supplementary Figure 5&6. Forest plot for subgroup analysis of AD and non-AD (MCI+NC) (AD: r= 0.4222, 95% CI:0.2372 to 0.5776, k=6; non-AD: r= 0.2728, 95% CI: 0.1494 to 0.3879, k=14; p=0.1735)
